# The Nature and Nurture of Congenital Amusia: A Twin Case Study

**DOI:** 10.3389/fnbeh.2018.00120

**Published:** 2018-06-25

**Authors:** Jasmin Pfeifer, Silke Hamann

**Affiliations:** ^1^Phonetics Laboratory, Amsterdam Center for Language and Communication, University of Amsterdam, Amsterdam, Netherlands; ^2^Institute for Language and Information, Heinrich-Heine University, Düsseldorf, Germany

**Keywords:** congenital amusia, twin study, pitch processing, spatial processing, hereditariness

## Abstract

In this article, we report the first documented case of congenital amusia in dizygotic twins. The female twin pair was 27 years old at the time of testing, with normal hearing and above average intelligence. Both had formal music lesson from the age of 8–12 and were exposed to music in their childhood. Using the Montreal Battery of Evaluation of Amusia (Peretz et al., 2003), one twin was diagnosed as amusic, with a pitch perception as well as a rhythm perception deficit, while the other twin had normal pitch and rhythm perception. We conducted a large battery of tests assessing the performance of the twins in music, pitch perception and memory, language perception and spatial processing. Both showed an identical albeit low pitch memory span of 3.5 tones and an impaired performance on a beat alignment task, yet the non-amusic twin outperformed the amusic twin in three other musical and all language related tasks. The twins also differed significantly in their performance on one of two spatial tasks (visualization), with the non-amusic twin outperforming the amusic twin (83% vs. 20% correct). The performance of the twins is also compared to normative samples of normal and amusic participants from other studies. This twin case study highlights that congenital amusia is not due to insufficient exposure to music in childhood: The exposure to music of the twin pair was as comparable as it can be for two individuals. This study also indicates that there is an association between amusia and a spatial processing deficit (see Douglas and Bilkey, 2007; contra Tillmann et al., 2010; Williamson et al., 2011) and that more research is needed in this area.

## Introduction

Congenital amusia is an innate disorder that has been shown to have a negative influence on pitch perception (Peretz et al., 2002; Foxton et al., 2004; Stewart, 2008), with a co-occurring deficit in rhythm perception in about 50% of the cases (Pfeifer and Hamann, 2015). This congenital variety of amusia is neither caused by a hearing deficiency nor by any form of brain damage or intellectual impairment (Ayotte et al., 2002) and causes persistent, lifelong impairments in the musical (Stewart, 2008), or more broadly, auditory domain. While congenital amusia had long been reported to affect only the musical domain (Peretz, 2001; Ayotte et al., 2002; Peretz et al., 2002), many recent studies have shown that different areas of speech perception are also affected, such as the perception of intonation (Patel et al., 2008; Liu et al., 2010; Hamann et al., 2012), of tone in languages that employ tone differences distinctively (Tillmann et al., 2011; Liu et al., 2012, 2015a,b), the perception of vowels (Huang et al., 2016; Zhang et al., 2017) and of emotional prosody in language (Thompson et al., 2012; Lolli et al., 2015).

The prevalence of the disorder is estimated to range between 1.5% (Peretz and Vuvan, 2017) and 4% (Kalmus and Fry, 1980) of the general population. Because of its clustering within families, documented in the first and so far only familial aggregation study by Peretz et al. (2007), congenital amusia has been hypothesized to have a genetic component. Peretz et al. (2007) studied 13 amusics from nine families and calculated a sibling recurrence risk ratio (the ratio of manifestation, given that a sibling is affected, compared with the prevalence in the general population; Risch, 1990) of λ_s_ = 10.8. This ratio is in the same order of magnitude as the heritability of specific language impairments and of absolute pitch. Based on these numbers, recent studies think it very likely that congenital amusia has a hereditary component (Peretz et al., 2007; Gingras et al., 2015; Peretz and Vuvan, 2017). However, familial aggregation could be simply due to shared family environment (in the case of congenital amusia, e.g., non-exposure to music within a family). Such environmental factors can only be reliably separated from genetic effects in twin studies, which have been employed successfully to test the heritability of pitch processing in general. Drayna et al. (2001), for instance, compared musically non-preselected monozygotic (*N* = 136) and dizygotic (*N* = 148) twin pairs using the Distorted Tunes Test (DTT; Kalmus and Fry, 1980) in a large-scale study. The heritability of pitch processing as estimated by their genetic model fitting was 71%, and they found a high correlation (0.67) in liability within monozygotic twin pairs and a medium one (0.44) for dizygotic twin pairs. A newer twin study on general pitch and rhythm perception (Seesjärvi et al., 2016) used three subtests from an online musicality test (Peretz et al., 2008) with 69 monozygotic and 44 dizygotic twin pairs to compare genetic and environmental effects. The correlations of scores within the twin pairs on the scale test was comparable to Drayna et al. (2001) with a high correlation (0.58) for monozygotic and a medium one (0.38) for dizygotic twin pairs. On the out-of-key test, a high correlation was found for both twin groups (0.63 monozygotic and 0.67 dizygotic) and on the off-beat test only a medium correlation (0.31) for monozygotic twin pairs. Mosing et al. (2014) tested a large sample of 2568 Swedish twins with a rhythm, a melody and a pitch task. They also found similarly high correlations of 0.57 for melody and 0.48 for pitch in monozygotic twins but lower correlations of 0.32 for melody and 0.29 for pitch in monozygotic twins.

A pitfall of utilizing such twin studies in congenital amusia research is the sample size. The recruitment of amusic participants in general is already difficult, while the recruitment of a sufficiently sized pool of amusic twin pairs is nearly impossible. Most amusia studies have small sample sizes, and some are single subject studies, e.g., Peretz et al. (2002) reporting the first case of amusia or Lebrun et al. (2012) reporting the first case of amusia in a child.

In the present study, we report the first documented case of congenital amusia in a dizygotic twin. With these twins, we conducted a large battery of tests assessing their musicality, pitch perception and pitch memory, language perception and spatial abilities in order to determine a possible genetic impact of amusia on these abilities. An overview can be found in Table 1. We chose to use not only the Montreal Battery of Evaluation of Amusia (MBEA; Peretz et al., 2003) to assess amusics’ music perception, as it has been criticized lately (Henry and McAuley, 2013; Pfeifer and Hamann, 2015) but also conducted the Goldsmith Musical Sophistication Index (GoldMSI, Müllensiefen et al., 2014). The Gold-MSI has never been conducted with amusics to our knowledge, so our twin pair will be compared to available norm samples. We thereby hope to obtain a broader perspective on the musical abilities and disabilities of our amusic twin in comparison to the non-amusic twin. We also included pitch perception tasks, as these are now widely used to determine amusics’ pitch thresholds, and memory span tasks to investigate possibly different memory capacities of the twins. In addition, we wanted to asses the twins’ language perception, as an increasing body of literature points to deficits in speech perception as well. We decided to also include tests on spatial abilities, as deficits in spatial processing by amusics have been found by Douglas and Bilkey (2007). Douglas and Bilkey used a classic Mental Rotation task (Shepard and Metzler, 1971) with line drawings of two three-dimensional objects that had to be compared, and amusics showed significantly higher error rates on this task. Later tests failed to replicate these findings. Tillmann et al. (2010) utilized the same Mental Rotation task but with 160 trials instead of the 20 employed by Douglas and Bilkey. In addition they also used a bisection task in which the midpoint of a straight line or a string of letters has to be marked. They found no difference between controls’ and amusics’ accuracy or reaction time on either task. Williamson et al. (2011) again used a version of the Mental Rotation task and two further tasks assessing memory for sequences of spatial location (Milner, 1971) and memory for visual patterns (Della Sala et al., 1997). No difference in accuracy between amusics (*N* = 14) and controls (*N* = 14) on any of these tasks was found. However, a subgroup of amusics with the most severe pitch perception deficits exhibited slower reaction times on the Mental Rotation task. Peretz and colleagues (Peretz et al., 2008; Peretz and Vuvan, 2017) report that amusia and visuo-spatial deficits are associated, though this is solely based on self-report questionnaire data.

**Table 1:** Overview of the assessed abilities and the utilized tests with references.

Ability	Task	Subtests	Reference
Musical	Goldsmith Musical Sophistication	Questionnaire	Müllensiefen et al. (2014), Schaal et al. (2014) and Fiedler and Müllensiefen (2015)
	Index (Gold-MSI)	Gold-Genre	
		Gold-Melody	
		Gold-BAT	
Pitch perception and memory	Pitch perception task	Detection	Williamson and Stewart (2010)
		Direction	
	Memory span task	Pitch Span	Williamson and Stewart (2010) and Schaal et al. (2015)
		Visual Span	
Language perception	Intonation task	Intonation	Hamann et al. (2012)
	Vowel perception task	Vowel	
Spatial	Object Perspective Taking Test	Orientation	Hegarty and Waller (2004)
	Santa Barbara Solids Test	Visualization	Cohen and Hegarty (2012)

## Materials and Methods

### Procedure

First, we assessed the twins with the Montreal Battery of Evaluation of Amusia (Peretz et al., 2003) and a questionnaire about educational, musical and demographic background. In addition, we assessed the twins’ hearing and their intelligence. In order to further ascertain the differences and similarities in their musical, pitch perception and memory, language and spatial abilities, we then conducted a number of additional tests, listed in Table 1.

All experiments were conducted at the University of Düsseldorf in the phonetics laboratory in a sound-insulated booth. All experiments were programmed in Praat (Boersma and Weenink, 2016) unless otherwise mentioned, and auditory stimuli were presented over AKG K 601 headphones on a windows XP computer. All data were collected in accordance with the declaration of Helsinki. Both participants gave informed written consent to participate in this study and received a small monetary reimbursement for their time. Both participants completed all test over the course of several days. The twins took the same tests on the same days, right after each other so that they did not have the possibility to exchange information on the tasks before both had completed them.

### Participants

The female twins were 27 years old at the time of testing with no history of psychiatric or hearing disorders. They grew up together in the same household with one younger male sibling and attended primary and secondary school and their undergraduate program in linguistics together. They had music lesson (flute) from the age of 8–12 and had the same exposure to music in their childhood and adolescence. The parents of the twins still live together. The mother does not show signs of amusia and seems to enjoy music. The father, however, has a severe hearing deficit in both ears that has been present since childhood due to a measles infection, and he uses hearing aids. He therefore had no normal exposure to music in childhood. Due to his severe hearing impairment, we could not test him for amusia and we cannot make any statement whether he might be amusic or not.

For the diagnosis of the twins, the MBEA (Peretz et al., 2003) and a questionnaire were used (the latter is described in detail in Pfeifer and Hamann, 2015: pages 9–11). Their scores on the MBEA are given in Table 2. One twin, called A in the following, falls below the cut-off scores by Peretz et al. (2003) on the first four subtests, exhibiting a pitch and a rhythm perception deficit. The other twin, called C in the following, stays well above the cut-off scores on all subtests. A further analysis of the MBEA results with signal detection theory (SDT; Green and Swets, 1966; MacMillan and Creelman, 2005) was carried out, as the SDT measure *d*′ is bias free and reflects participants’ discriminatory ability without the response bias. The twins show clearly distinct discriminatory abilities, with C having much higher scores i.e., being able to discriminate much better between stimuli than A in all but the Meter subtest, where A is slightly better than her non-amusic twin sister. The *d*′ scores for the Meter subtest are rather low for both twins, which reflects the problematic nature of this subtest (see Pfeifer and Hamann, 2015, for details).

**Table 2:** Montreal Battery of Evaluation of Amusia (MBEA) scores of the twins based on sum of correct responses out of 30 where cut-off score by Peretz et al. (2003) are given in brackets, and *d*′ scores.

	Scale	Contour	Interval	Rhythm	Meter	Memory
Sum correct responses A	20 (22)	21 (22)	20 (21)	21 (23)	25 (20)	26 (22)
Sum correct responses C	27 (22)	26 (22)	26 (21)	29 (23)	24 (20)	28 (22)
*d*′ A	1.8	2.07	1.25	1.42	1.95	2.34
*d*′ C	3.07	2.95	2.95	3.83	1.68	3.44

The answers to the questionnaire confirmed the results obtained by the MBEA.

Both twins have normal hearing defined as a mean hearing level of 20 dB or less in both ears (tested with a pure tone audiometry at 250, 500, 1000, 2000, 3000, 4000, 6000 and 8000 Hz). The twins intelligence was assessed using the German version of the Hamburger Wechsler Adult Intelligence Scale (HAWIE; Wechsler, 1964). The twins both exhibited higher than average intelligence scores belonging to the highest 2% of scores. The non-amusic twin C achieved a global score of 132 IQ points (verbal 111, action 139) and the amusic twin A a global score of 138 (verbal 124, action 136) IQ points. Both reached similar scores on all subtests with the exception of the digit span subtest, where A had problems in comparison to her twin.

### Further Musical Abilities

In addition to the MBEA, we also employed the Goldsmith Musical Sophistication Index (Müllensiefen et al., 2014), to further assess the musical performance of our twin pair. We tested them with four of the five parts of the Gold-MSI: A self-report questionnaire (the German version hereof, see Schaal et al., 2014; Fiedler and Müllensiefen, 2015), a genre sorting task (Gold-Genre), a melody memory task (Gold-Melody), and a beat alignment perception task (Gold-BAT). The Gold-Genre task consists of 16 musical excerpts, each 800 ms long, without lyrics or vocals. The excerpts are taken from four different genres (pop, rock, jazz and hip-hop) and participants have to group them into four categories without being told what the categories are. The Gold-Melody task consists of 13 melody pairs that have to be compared. Each melody is between 10 and 16 notes long, and the second melody is always transposed to a different key to test memory for a melody’s interval structure rather than absolute pitch. The two melodies are either the same—except for the key transposition of which subjects are informed—or the second melody contains an alteration. The Gold-BAT task is based on the Beat Alignment Test by Iversen and Patel (2008) and investigates beat-based processing. The test consists of 12 melodies from three different genres, and a beat track is superimposed on every melody. The participant’s task is to judge whether the beat track is on the beat of the music or not.

### Pitch Perception and Pitch Memory Abilities

We employed two tasks previously used by Williamson and Stewart (2010) to investigate the auditory pitch perception abilities of our two participants. The pitch detection task measures the threshold for the detection of a pitch change, while the pitch direction task measures the threshold for discriminating pitch direction. Both are two-alternative forced choice AXB tasks employing an adaptive two-up-one-down staircase procedure. Every trial consisted of three consecutive tones, each 600 ms long. In the pitch detection task, the target tone was a pitch glide centered around 500 Hz, while the two non-target tones were steady-state tones with a frequency of 500 Hz. In the pitch direction task, all three tones were pitch glides centered around 500 Hz. The target tone was a glide in the opposite direction to the two non-target tones. The task was to identify which tone was different: the first or the last. Each task started with a pitch difference of six semitones. When participants gave two consecutive correct answers, they advanced a level, and the pitch difference became smaller. When they made one mistake, they went one level down and the pitch distance became larger. Each task ended after 15 level changes. To increase the precision of threshold determination, variable pitch step sizes were used. For the first five level changes, the change consisted of one semitone. For level changes 6–9, a change of 0.2 semitones was used, and for levels 10–15 a change of 0.05 semitones. The last 10 trials were averaged to compute the perceptual threshold of the participants.

We also included a test assessing participants’ short-term memory for auditory as well as visual sequences with a two-alternative forced choice design (Williamson and Stewart, 2010; Schaal et al., 2015). The auditory stimuli were 10 sine wave tones with a duration of 500 ms and with fundamental frequencies ranging from 262 Hz to 741 Hz in whole tone steps. The visual stimuli were 10 Devanagari letters presented for 500 ms in black on a white background. The procedure was the same for both types of stimuli: 500 ms of silence or a blank screen were followed by two successive, equally long sequences of tones or letters. The two sequences in a trial were either identical or the position of two tones/visual signs was switched in one of the sequences. The participants’ task was to determine whether the two sequences were identical or different. The same two-up-one-down staircase procedure described above for the pitch perception thresholds was employed, and the difficulty advanced, i.e., the sequences became longer after two consecutive correct answers and shorter after one incorrect answer. Each task was terminated after four incorrect answers. The last 10 trials of each task were used to calculate participants’ memory span, indicating the (auditory or visual) memory load they can store in each domain.

### Language Perception Abilities

#### Intonation Perception

To test the intonation perception of our twin pair, we used the AX same-different discrimination task and stimuli from Hamann et al. (2012), which was in turn based on the study by Patel et al. (2008). The stimuli pairs were based on recordings of four German statement-question pairs spoken by a male native speaker. Each pair was identical but for the final intonation contour, i.e., statements exhibiting a falling pattern and the corresponding echo questions a rising pattern. The intonation contour of questions was manipulated downwards in seven steps of one semitone each, while the intonation contour of questions was manipulated upwards in the same way. Stimulus pairs consisted of the original statement or question followed either by one of the downward or upward manipulations or the original again, resulting in 112 stimuli pairs. Participants had to indicate for each pair whether the two were identical or not. We also included sinusoidal wave analogs (similar to Patel et al., 2008) that did not contain any linguistic material but were solely based on the intonation contour of the speech stimuli. These were manipulated and paired in the same way as the speech stimuli. The test was scored by calculating three different performance measures: hit rate, percentage correct and *d*′. Hit rate is solely based on answers to stimulus pairs where A differs from X, which are considered a hit when they are correctly identified as different. Percentage correct is the sum of both hits and correct rejections (stimulus pairs where A and X are the same and which are correctly identified as same) in relation to all answers.

#### Vowel Perception

**Figure 1:**
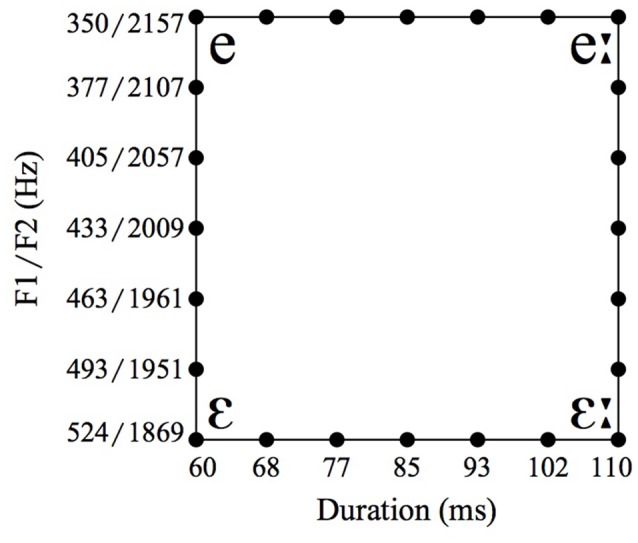
Spectral and durational values of vowel stimuli.

The second language-related task consists of an AXB forced-choice discrimination task with vowel stimuli. We used isolated synthetic vowels based on auditory properties of the natural German vowels /ε/ and /e:/, where /e:/ is 110 ms long with a first formant (F1) of 350 Hz and a second formant (F2) of 2157 Hz, and /ε/ 60 ms long with an F1 of 524 Hz and an F2 of 1869 Hz (based on Jessen, 1993). On the basis of these vowels we created four continua with seven steps each, depicted as the four sides of the rectangular in Figure 1. For each AXB trial, A and B were the endpoints of one continuum (one side of the rectangular), and X could either be one of the two endpoints or one of the five vowels in-between. The trials were offered with two different inter-stimulus intervals (ISIs) of either 0.2 s or 1.2 s (Werker and Logan, 1985; Williamson and Stewart, 2010). Each trial was repeated five times throughout the experiment.

The vowel perception task was scored by calculating the percentage of how often participants perceived X correctly as category A (where the answer was considered correct when X was either identical to A or one of the three stimuli close to A on the continuum in question). Based on this measure we calculated *d*′ values.

### Spatial Abilities

The Mental Rotation task used in previous studies to test amusics’ spatial abilities has been argued to be rather complex and to rely on different cognitive processes (Williamson et al., 2011), we therefore decided to employ the Object Perspective Taking Test (Hegarty and Waller, 2004) and the Santa Barbara Solids Test (Cohen and Hegarty, 2012) instead. These two tests were chosen as they differentiate between spatial orientation abilities, tested with the Object Perspective Taking Test, and spatial visualization abilities, tested with the Santa Barbara Solids Test.

In the Object Perspective Taking Test, the participant is asked to imagine the degree in which several objects are placed to each other from different perspectives, providing a test of egocentric spatial transformations. The test was administered in a paper-and-pencil based version and contained 12 items. Each item consists of a map in the top half of the page, in which seven items are arranged. Participants are asked to imagine being at the position of one object, facing a second one, and having to point to a third object. On the bottom of the page is a circle and the first object is always located in its center with an arrow pointing vertically up to the second object. Participants have to draw a second arrow from the center of the circle outwards to the position of the third target object, thereby making an egocentric transformation. Participants are prevented from rotating the paper, so as not to make the task easier. The perspective change on every item is at least 90 degrees. Each item is scored by calculating the deviation from the correct direction in degrees. The overall score on the test is the average deviation across all items.

**Figure 2:**
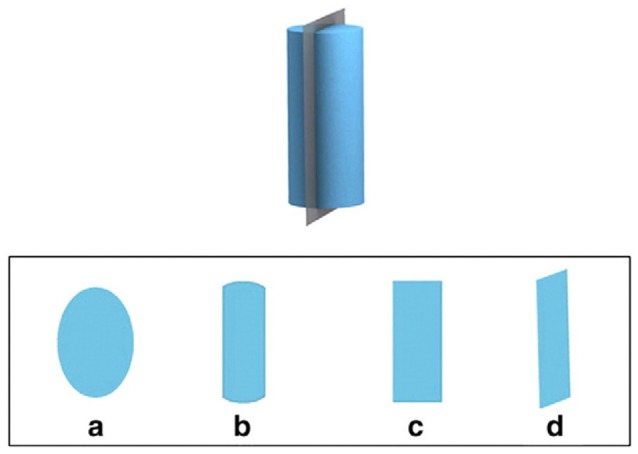
Example item from the Santa Barbara Solids Test (Cohen and Hegarty, 2012: 869). The top depicts a three-dimensional object and a plane cutting this object vertically, the bottom displays four cross-sections as answer choices ((c) being the correct answer, and (d) the distracter without change in view perspective).

The Santa Barbara Solids Test was also administered in a paper-and-pencil version containing 30 items. Each item consists of a three-dimensional geometric object that is sliced by a plane. Participants are asked to imagine looking at the two-dimensional cross-section of the geometric object caused by the plane. The stimuli vary in complexity along two factors: Complexity of the geometric shape and the orientation of the cutting plane. Half of the items have planes that are vertical or horizontal to the main axis of the shape, and the other half have planes that are diagonal to this axis. Participants are given four answer choices, depicted as possible cross-sections. The answers include one egocentric distracter that represents the shape that a participant who fails to change her perspective would choose, providing a way to differentiate whether a perspective change away from egocentric was made or not; see the example in Figure 2.

The Santa Barbara Solids Test is scored by counting the number of correct responses and calculating the percentage correct.

## Results

The pitch perception and memory tasks as well as the language perception tasks have previously been used with amusics, and the performance of the twin pair is compared to those samples. The Gold-MSI has never been conducted with amusics, therefore no cut-off scores for amusics are available. However, Müllensiefen et al. (2014) provide data norms based on 147,636 participants, to which we compared our two subjects. Similarly, the spatial tasks have not been administered to amusics before, and we compared the twins’ performance to the data norms by Hegarty and Waller (2004) for the Object Perspective Taking Test (based on 62 participants) and the norms by Cohen and Hegarty (2012) for the Santa Barbara Solids Test (223 participants).

### Further Musical Abilities

The Gold-MSI questionnaire yields six factors, which can be found in Table 3 with mean scores and endpoint of scales from Müllensiefen et al. (2014). A’s scores are almost exclusively situated in the lowest percentile in all factors but the musical education one, due to both twins having had music lessons for 4 years. C’s scores are higher and range between the 2nd and 40th percentile.

**Table 3:** Scores on Gold-MSI questionnaire, where numbers in brackets denote percentile of score.

	Active engagement	Perceptual abilities	Musical training	Singing abilities	Emotions	General musical sophistication
A	9 (1)	22 (1)	11 (11–13)	8 (1)	12 (1)	21 (1)
C	19 (2)	48 (37–40)	12 (14–15)	24 (18–19)	32 (26–31)	51 (8)
Scale Min	9	9	7	7	6	18
Scale Max	63	63	49	49	42	126
Mean	41.52	50.20	26.52	31.67	34.66	81.58
SD	10.36	7.86	11.44	8.72	5.04	20.62

*Lower part of the table: Norms based on 147,633 participants from Müllensiefen et al. (2014)*.

The results of the Gold-Genre task are scored as total correct pairs (of two) per participant. Every possible pair in every classification group is counted. This way, a total of 24 pairs is possible. Twin A scored 4 out of 24 possible pairs (15th percentile) and twin C scored seven pairs (44th–53rd percentile). A’s performance was thus again rather low (comparable to her performance on the questionnaire), while C performed better than her twin.

The Gold-Melody task can be scored using either accuracy or *d*′. Twin A obtained an accuracy score of 0.69 (36th–40th percentile) and a *d*′ score of 0.93 (26th–30th percentile). Twin C performed well with an accuracy score of 0.85 (76th–80th percentile) and a *d*′ score of 2.58 (76th–80th percentile).

The Gold-BAT task can again be scored using either accuracy or *d*′. Twin A obtained an accuracy score of 0.47 (1st–5th percentile) and a *d*′ score of 0.38 (16th–20th percentile). Twin C obtained an accuracy score of 0.53 (6th–10th percentile) and a *d*′ score of 0.58 (21st–25th percentile), thus neither of them performed well on the beat alignment subtest. Corroborating results were obtained with the perceptual part of the Beat Alignment Test by Iversen and Patel (2008), with which we additionally tested the twins. Since part of this test is identical to Gold-BAT and no scores of amusics are available for comparison, we refrain from reporting the detailed results.

### Pitch Perception and Pitch Memory Abilities

For the pitch detection task, both A and C had thresholds of 0.14 semitones, which is comparable to the value Williamson and Stewart (2010) found for their control group. The threshold for their amusic group was slightly, however not significantly, higher, see first row in Table 4.

**Table 4:** Results of pitch detection and pitch direction task and results from Williamson and Stewart (2010) and Schaal et al. (2015) for comparison, all in semitones.

Task	Twins		Williamson and Stewart (2010)		Schaal et al. (2015)	
	A	C	Amusic *N* = 14	Control *N* = 14	Amusic *N* = 8	Control *N* = 8
Pitch detection threshold	0.14	0.14	0.28 (±0.18)	0.14 (±0.03)	0.44 (±0.22)	0.36 (±0.22)
Pitch direction threshold	0.55	0.15	0.95 (±0.50)	0.17 (±0.05)	0.89 (±0.31)	0.19 (±0.03)

*Values in brackets indicate 95% confidence interval*.

With respect to pitch direction discrimination, twin A reached a threshold of 0.55 semitones, which is considerably higher than her sister’s: twin C had a threshold of 0.15 semitones, again comparable to the values of Williamson and Stewart (2010) and also to those by Schaal et al. (2015), see second row in Table 4.

In the memory span task, both twins had comparatively low pitch spans of 3.5 and 3.3 tones respectively, which is comparable to the amusics’ results in the previous studies, see first row in Table 5. Interestingly, there was a substantial difference in the performance of the two twins for the visual memory task: A had a visual memory span of only 2.2 letters, while C had a visual memory span of 8.8 letters. This is in contrast to Williamson and Stewart (2010) and Schaal et al. (2015) data, in which the amusics did not differ significantly from the controls in their visual memory span, see second row in Table 5.

**Table 5:** Results of auditory memory span task (in tones) and visual memory span task (in letters), and for comparison the results from Williamson and Stewart (2010) and Schaal et al. (2015).

Task	Twins		Schaal et al. (2015)		Williamson and Stewart (2010)	
	A	C	Amusic *N* = 8	Control *N* = 8	Amusic *N* = 14	Control *N* = 14
Pitch Span	3.3	3.5	3.94	5.4	4.13	6.80
Visual Span	2.2	8.8	5.92	6.82	6.88	7.57

*Schaal et al. (2015) employed the same visual stimuli (Devanagari letters) as the present study, while Williamson and Stewart (2010) used digits instead*.

### Language Perception Abilities

#### Intonation Perception

The performance measures hit rate, percentage correct and *d*′ for the intonation perception task are given in Table 6. Both twins differ on all three, and exhibit comparable performance to Hamann et al. (2012) cohort of amusics and controls respectively. For further analysis of the different semitone interval steps, only *d*′ values were calculated. Table 7 displays these *d*′ values for the speech stimuli only and for the combination of both speech and sine analog stimuli. The latter is comparable to the results by Hamann et al. (2012) given in the last column of the table. As Table 7 shows, the *d*′ values by A and hence her discriminatory ability were consistently lower at all interval sizes in comparison to her twin C.

**Table 6:** Results of intonation task cumulated across all data (speech and sine analog together) and Hamann et al.’s (2012) for comparison.

Performance measure	Twins		Hamann et al. (2012)	
	A	C	Amusics	Controls
			*N* = 7	*N* = 35
Hit rate	0.26	0.67	0.48 (±0.15)	0.78 (±0.46)
Percentage correct	62.05	80.80	68.58 (±5.75)	81.86 (±20.82)
d′	1.45	2.05	1.28 (±0.27)	2.00 (±1.17)

*Values in brackets indicate 95% confidence interval*.

**Table 7:** *d*′ values of C and A for each interval size for the speech stimuli and for both speech and sine analog stimuli together, and for comparison *d*′ values from Hamann et al. (2012) across all of their data (speech, sine analog and pulse train analog).

Interval	Speech only		All data		Hamann et al. (2012)	
	Twin A	Twin C	Twin A	Twin C	Amusics	Controls
1	0.00	0.65	−0.23	0.46	0.49	0.66
2	0.00	1.48	−0.23	1.12	0.51	1.42
3	0.00	2.48	−0.23	2.10	0.96	1.88
4	2.64	2.48	1.94	2.29	1.29	2.12
5	2.01	2.95	1.61	2.50	1.44	2.53
6	2.33	4.13	2.10	3.94	1.72	2.63
7	3.00	4.13	2.26	3.94	1.88	2.90

The perceptual thresholds for A and C were calculated (for speech and sine analogs together) as elaborated in Hamann et al. (2012), resulting in a perceptual threshold of 5.39 semitones for A and 1.91 semitones for C. The thresholds for the speech stimuli, only, were 6.01 semitones for A and 2.25 semitones for C. This indicates that A’s perception only reaches above chance performance at a difference of more than 5 semitones and is impaired in comparison to her sister’s. These values are comparable to (though higher than) Hamann et al.’s (2012) findings of 3.80 semitones for their amusic group and 1.67 semitones for their control group.

#### Vowel Perception

**Figure 3:**
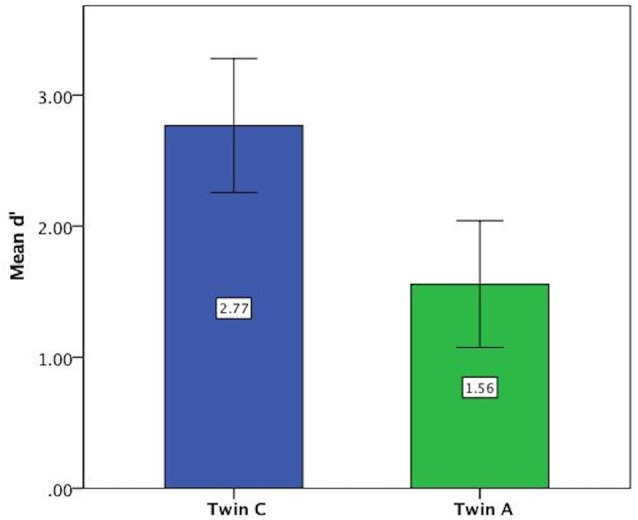
Mean *d*′ values on vowel task showing lower discriminatory ability of Twin A. Error bars: 95% CI.

**Figure 4:**
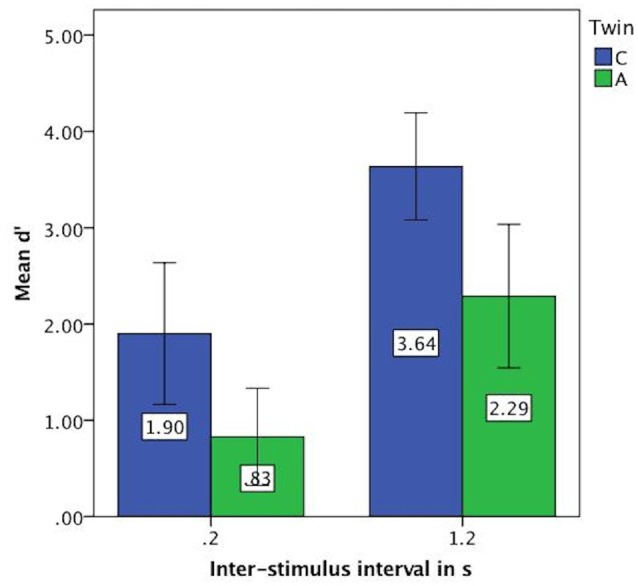
Mean *d*′ values on vowel task split by inter-stimulus interval (ISI) showing lower discriminatory ability for both twins at 0.2 s. Error bars: 95% CI.

For the vowel perception task, twin A had lower *d*′ values on average and thus a lower discriminatory ability (Mean = 1.56, SE = 0.24) than twin C (Mean = 2.77, SE = 0.25), see Figure 3. This difference was significant at *t*_(94)_ = 3.46, *p* = 0.001. Further analysis revealed that both twins have more difficulty with the shorter ISI of 0.2 s, as shown by lower discriminatory abilities (*d*′ values) in Figure 4.

**Figure 5:**
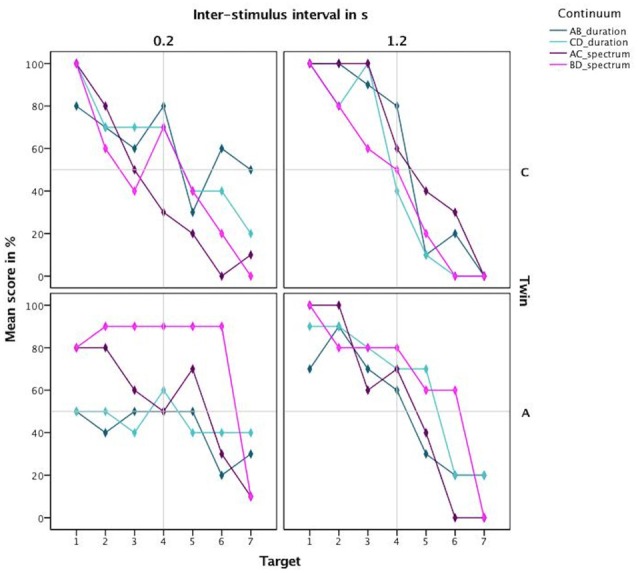
Discrimination curves based on mean scores in percent for both twins highlighting the different categorization boundary for the amusic twin for spectral cues.

Next, based on the percentage correct, we calculated a discrimination curve per continuum per participant, further split by the ISI. The resulting discrimination curves are visible in Figure 5. The steep discrimination curves for the longer ISI of 1.2 s, displayed in the right panel, show a clear categorization boundary. C’s boundary is located between the 3rd and the 5th stimulus for all continua, which is to be expected. A’s boundary is located between the 3rd and the 5th stimulus for the durational continua and between the 2nd and the 4th for the spectral continua. For the short inter-stimulus-interval of 0.2 s, the curves are not as steep in the boundary region. Especially A does not exhibit a steep slope for the durational continua.

### Spatial Abilities

On the Object Perspective Taking Test, Twin A had a higher degree of deviation than twin C, namely 33.25° compared to 24.58°. The scores of both twins are above the sample mean of 24.53 by Hegarty and Waller (2004); and do not differ from each other significantly, as shown by an independent samples *t*-test *t*_(22)_ = −0.577, *p* > 0.05.

On the Santa Barbara Solids Test, A achieved only a score of 20% correct answers, while C scored 83% correct answers and is above the sample mean of 68% provided by Cohen and Hegarty (2012). With respect to mistakes that show a failure in change of perspective, A made 50% and C 7% of such egocentric mistakes; the sample mean by Cohen and Hegarty is 19%.

There are no cut-off scores given for either of the two tasks. However, if two standard deviations are subtracted from the mean and this is taken as a cut-off score, than A’s performance is still clearly an outlier on the Santa Barbara Solids Test, see Table 8.

**Table 8:** Results of twins on Object Perspective Taking Test (Hegarty and Waller, 2004) and the Santa Barbara Solids Test (SBST; Cohen and Hegarty, 2012) with norm values.

Test	Twin A	Twin C	Hegarty and Waller (2004) *N* = 62	Cohen and Hegarty (2012) *N* = 223
Object Perspective Taking Test mean degree of deviation	33.25	24.58	24.53 SD 14.29	
Santa Barbara Solids Test score in absolute numbers out of 30	6 (15)	25 (2)		
Santa Barbara Solids Test score in percent	20% (50%)	83% (7%)		68% (19%) SD 23% (11%)

*Value in brackets on the SBST indicates egocentric transformation mistakes*.

## Discussion

In this twin case study, we tested a dizygotic twin pair with one amusic twin and one non-amusic twin. Both twins had normal hearing and above average intellectual abilities, the latter also reflecting their higher than average education, both being graduate students at the time of testing (Asendorpf, 2009). Musical exposure and education of the twins was as comparable as it can be for two individuals, we can therefore conclude that congenital amusia is not due to differences in musical education or to insufficient exposure to music in childhood or adolescences as previously discussed by e.g., Peretz (2001).

A comprehensive overview of the twins’ abilities as tested in this study is given in Table 9.

**Table 9:** Overview of assessed abilities and results per twin.

Ability	Task	Twin A (amusic)	Twin C (non-amusic)
Musical	Questionnaire	impaired	(√)
	Gold-Genre	impaired	√
	Gold-Melody	impaired	√
	Gold-BAT	impaired	impaired
Pitch perception and memory	Detection	√	√
	Direction	impaired	√
	Pitch Span	impaired	impaired
	Visual Span	impaired	√
Language perception	Intonation	impaired	√
	Vowel	impaired	√
Spatial	Orientation	√	√
	Visualization	impaired	√

Besides the MBEA (Peretz et al., 2003), which clearly diagnosed one twin as amusic and the other as non-amusic, we employed the Goldsmith Musical Sophistication Index (Müllensiefen et al., 2014) to test their musical abilities further. Its self-report questionnaire reflects both twins’ comparably low musical education (4 years), but still clearly differentiates the twins, with the amusic twin always scoring in the lowest percentile. A slight exception is the factor of Active Engagement, where both twins score in the lowest two percentiles. Clear differences for the twins also emerge on the Gold-Genre and Gold-Melody subtests, with the non-amusic twin outperforming the amusic one. Only on the Gold-BAT subtest is their performance very similar and in a rather low range. This finding is not in line with the performance of the non-amusic twin on the MBEA Rhythm subtest, which was very high (her highest score on any of the subtests), while the amusics’ score was very low. A larger study of the Gold-MSI with amusics should be conducted in the future to see whether the pattern shown by the amusic in this study holds for a larger group of amusics, i.e., whether the Gold-MSI can be used to reliably differentiate amusics from non-amusics. In addition, the MBEA is very repetitive and tedious to complete for amusics, while the Gold-MSI offers different tasks and has a questionnaire already included. So, future directions might be to use the Gold-MSI in addition to the MBEA or possibly even as a replacement, since the MBEA has an imbalance in pitch and rhythm-based subtests, as was already pointed out by Pfeifer and Hamann (2015).

The finding that both twins have a comparable low pitch detection threshold of 0.135 tones (indicating no impairment), while their pitch direction threshold differs, is in line with previous findings on amusics (Williamson and Stewart, 2010), and indicates that their auditory processing is unimpaired but that congenital amusia has an impact on the perception of changes in pitch direction. It is surprising that both twins exhibit a low pitch memory span in comparison to normal controls (Schaal et al., 2015), which might be interpreted as an indication for a certain hereditariness of pitch memory, as has been proposed for pitch processing (Drayna et al., 2001). Mosing et al. (2014) report a positive association for their large twin cohort between the different auditory tasks for the twin pairs. They find that this is mostly due to shared genes and to a smaller degree to shared environmental factors affecting musical abilities. This leads back to a nature vs. nurture debate and ties into the question of the genetic underpinnings of congenital amusia. The dizygotic twin pair share 50% of their genes and we can assume that congenital amusia—since it is only present in one twin—is somehow encoded in the 50% of non-shared genes. What is puzzling in the present case, however, is that both twins exhibit pitch memory impairments. These could either be due to their 50% of shared genes or to their shared environment. In the future, gene sequencing of congenital amusia is required to unravel the underpinnings of this disorder and to further understand the genetics of musical abilities and general auditory processing. The dizygotic twin pair discussed in this article and a further amusic monozygotic twin pair that we have just identified seem to be a promising starting point for a genetic analysis.

While the everyday communication of the amusic twin seems to be unimpaired and her score on the verbal subscale of the Hamburger Wechsler Adult Intelligence Scale is high (124 IQ points compared to 111 by the non-amusic twin), her intonation perception and vowel perception are impaired in comparison to her sister, and she shows overall lower discriminatory abilities. This was to be expected based on previous studies on language perception by amusics (e.g., Liu et al., 2010; Hamann et al., 2012). Interestingly, the stimuli without linguistic information on the intonation perception task resulted in better performance for both twins than real speech stimuli. Future studies with amusics and controls need to test whether the presence of linguistic in addition to tonal information does not enhance pitch perception. On the vowel perception task, A exhibited a different categorical boundary for spectral cues than her twin. We are currently conducting a study on vowel perception with a larger pool of amusics and controls in order to investigate the pattern exhibited by the twins.

Lastly and most surprisingly, the twins also performed differently on one of the spatial tasks, with the non-amusic twin (83% correct) outperforming the amusic twin (20% correct). Taken together, the results indicate that the amusic twin can perform egocentric spatial transformations, as shown by the Object Perspective Taking Test, but struggles with object-based spatial transformations that were required in the Santa Barbara Solids Test. Her sister had no difficulties with the latter. This shows that at least this one amusic has impaired spatial visualization abilities with intact spatial orientation abilities. Our finding contrasts with that by Tillmann et al. (2010) who assume spatial abilities by amusics to be unimpaired based on their test, but are in line with Douglas and Bilkey (2007) finding, the self-reports given in Peretz and Vuvan (2017) and the longer reaction time latencies found by Williamson et al. (2011) for a subgroup of amusics. These indications for a very specialized impairment warrant further scrutiny of amusics’ spatial abilities and a fractionating of their skills in this regard.

## Conclusion

This study was the first to employ the Goldsmith Musical Sophistication Index to test the differences between an amusic and a non-amusic participant. All in all, the Gold-MSI seems to be able—at least in this very limited sample—to differentiate between non-amusic and amusic participants. In the future, a larger sample of amusics should be tested with it to asses whether this holds true for a larger group. If this is the case, the Gold-MSI could be used to supplement or possibly replace the MBEA in the diagnosis of congenital amusia in the future.

We also showed that the question of a spatial processing deficit in amusia needs to be revisited and more research is needed in that area. Most notably, separate tests should be employed for egocentric and object-based spatial transformations to be able to differentiate between the two, as only the latter turned out to be impaired in our amusic twin.

This twin case study highlights that congenital amusia is not due to insufficient exposure to music in childhood. The exposure to music of the twin pair was as comparable as it can be for two individuals. Yet, one twin has amusia, while the other does not. In addition to the expected differences in melodic and language perception abilities, we found that both twins exhibit a comparably low pitch memory span and low beat perception abilities. This raises the question of nature vs. nurture, i.e., whether their shared genes or their shared environment and low musical education is responsible for the shared deviant performance. This in turn gives rise to the question of hereditariness of congenital amusia and calls for a genetic analysis of affected individuals. To prove that genetic causes play a role in congenital amusia, a large-scale genetic analysis of amusics and their unaffected relatives is necessary. From such a study, we could learn more about how amusia can develop and could identify which genes contribute to higher cognitive functions of auditory perception.

## Author Contributions

JP posed the research question, recruited and tested the participants, chose and performed the statistical analyses and wrote a first draft of the manuscript. Both JP and SH designed the experiments together, discussed and interpreted the results together, and together restructured and rewrote the text.

## Conflict of Interest Statement

The authors declare that the research was conducted in the absence of any commercial or financial relationships that could be construed as a potential conflict of interest.
